# Functional changes in cytotoxic CD8+ T-cell cross-reactivity against the SARS-CoV-2 Omicron variant after mRNA vaccination

**DOI:** 10.3389/fimmu.2022.1081047

**Published:** 2023-01-04

**Authors:** Takuto Nogimori, Koichiro Suzuki, Yuji Masuta, Ayaka Washizaki, Mika Yagoto, Mami Ikeda, Yuki Katayama, Hidenori Kanda, Minoru Takada, Shohei Minami, Takeshi Kobayashi, Shokichi Takahama, Yasuo Yoshioka, Takuya Yamamoto

**Affiliations:** ^1^Laboratory of Immunosenescence, Center for Vaccine and Adjuvant Research, National Institutes of Biomedical Innovation, Health and Nutrition, Osaka, Japan; ^2^Research Institute for Microbial Diseases, Osaka University, Osaka, Japan; ^3^The Research Foundation for Microbial Diseases of Osaka University (BIKEN), Osaka, Japan; ^4^Laboratory of Aging and Immune Regulation, Graduate School of Pharmaceutical Sciences, Osaka University, Osaka, Japan; ^5^KINSHUKAI, Hanwa Memorial Hospital, Osaka, Japan; ^6^KINSHUKAI, Hanwa The Second Senboku Hospital, Osaka, Japan; ^7^Department of Virology, Research Institute for Microbial Diseases, Osaka University, Osaka, Japan; ^8^Vaccine Creation Group, BIKEN Innovative Vaccine Research Alliance Laboratories, Research Institute for Microbial Diseases, Osaka University, Osaka, Japan; ^9^Laboratory of Nano-design for innovative drug development, Graduate School of Pharmaceutical Sciences, Osaka University, Osaka, Japan; ^10^Institute for Open and Transdisciplinary Research Initiatives, Osaka University, Osaka, Japan; ^11^Department of Virology and Immunology, Graduate School of Medicine, Osaka University, Osaka, Japan

**Keywords:** COVID-19, SARS-CoV-2, mRNA vaccine, cytotoxic T cells, TCR repertoire

## Abstract

Understanding the T-cell responses involved in inhibiting COVID-19 severity is crucial for developing new therapeutic and vaccine strategies. Here, we characterized SARS-CoV-2 spike-specific CD8^+^ T cells in vaccinees longitudinally. The BNT162b2 mRNA vaccine can induce spike-specific CD8^+^ T cells cross-reacting to BA.1, whereas the T-cell receptor (TCR) repertoire usages decreased with time. Furthermore the mRNA vaccine induced spike-specific CD8^+^ T cells subpopulation expressing Granzyme A (GZMA), Granzyme B (GZMB) and Perforin simultaneously in healthy donors at 4 weeks after the second vaccination. The induced subpopulation was not maintained at 12 weeks after the second vaccination. Incorporating factors that efficiently induce CD8^+^ T cells with highly cytotoxic activity could improve future vaccine efficacy against such variants.

## Introduction

COVID-19, an infectious disease caused by severe acute respiratory syndrome coronavirus 2 (SARS-CoV-2), is an ongoing global pandemic with increasing incidence and mortality (1). SARS-CoV-2 infection causes a wide variety of clinical features, ranging from asymptomatic cases to severe cases with an inflammatory response and death (2, 3). The mortality rate of COVID-19 is lower than that of severe acute respiratory syndrome coronavirus (SARS-CoV) and Middle East respiratory syndrome coronavirus (MERS-CoV), but much higher than that of seasonal influenza (4, 5). The emergence of new variants of concern (VoCs) may further increase disease severity. The Delta strain, B.1.617.2 (first detected in India in December 2020), reduces vaccine efficacy (6). B.1.1.529 (the Omicron strain, which emerged in South Africa in November 2021 (7)) has many amino acid mutations in its spike protein, and 15 mutations in the ACE2 receptor-binding domain (8). Compared with previous VoCs, Omicron more severely reduces vaccine and monoclonal antibody efficacy (8–12). Furthermore, owing to its higher transmissibility, there are concerns about faster and wider spread of SARS-CoV-2 infection (13). Subsequently, strain BA.2, derived from the Omicron strain, has been detected worldwide since January 2022. The growth rate of BA.2 in the human population is 1.4-fold higher than that of the Omicron BA.1 strain. Antibodies induced by Omicron BA.1 have reduced neutralizing activity against Omicron BA.2, suggesting that the Omicron BA.1 and Omicron BA.2 strains have different antigenic properties (14). Furthermore, among the multiple sub-strains of the Omicron BA.2 strain that emerged, the replacement to the BA.5 strain was rapid worldwide. Omicron BA.5 shows 1.4-fold higher effective reproduction in the human population compared with Omicron BA.2, and is more resistant to neutralizing antibodies induced by infection or three vaccinations compared with Omicron BA.2 and other sub-strains (15). Thus, SARS-CoV-2 efficiently escapes humoral immunity by being rapidly mutated.

T cells are critical in eliminating many respiratory tract infections in the acute phase (16). Cytotoxic CD8^+^ T cells, which play a protective role in immune surveillance against viral infection, are an important component of vaccine-induced protective immunity (17). The induction of cellular and humoral immunity in response to COVID-19 is important in suppressing symptom severity (18, 19). Lymphopenia is associated with COVID-19 disease severity (20) and the depletion of CD8^+^ T cells, but not CD4^+^ T cells, and is associated with poor prognosis of COVID-19 patients (21). In SARS-CoV-2-infected ACE2-transgenic mice with depleted CD8^+^ T-cell population, the viral load in the lungs was elevated 5 days post infection (22), suggesting that CD8^+^ T cells play an important role in the early clearance of SARS-CoV-2. In rhesus monkeys with prior SARS-CoV-2 infection, CD8^+^ T cell depletion reduced their resistance to SARS-CoV-2 re-challenge (23). A recent study of HLA A*0201-restricted T cell responses reported that introducing a point mutation within S_269-277_ (YLQPRTFLL), a major epitope for the spike antigen, markedly reduced the reactivity of CD8^+^ T cells derived from infected and mRNA vaccine donors (24). Namely, it has been demonstrated that SARS-CoV-2 can escape antigen-specific CD8^+^ T cells targeting the most common epitope of the spike, which is restricted by HLA-class I, even with a single amino acid mutation within the spike protein. Although CD8^+^ T cells with these characteristic T-cell receptor (TCRs) have been analyzed epitope-specifically using MHC multimers, there is no preceding TCR repertoire analysis on TCR clonotypes of specific HLA class-I restricted spike-specific CD8^+^ T cell responses, including responses to mutant strain spike antigens (25, 26).

Analysis of SARS-CoV-2-specific T-cell responses in asymptomatic and symptomatic SARS-CoV-2-infected individuals revealed equivalent frequencies of antigen-specific T cells, although asymptomatic individuals had elevated IFN-γ and IL-2 production. While antigen-specific T-cell IFN-γ production and IL-10 and pro-inflammatory cytokine (IL-6, TNF-α and IL-1β) production were proportionate in asymptomatic individuals, their secretion was disproportionate in symptomatic individuals (27). In a longitudinal study of SARS-CoV-2-infected patients, from onset to recovery or death, IFN-γ-secreting SARS-CoV-2-specific T cells were induced earlier in individuals with mild disease than in those with severe disease (19). Indeed, early functional induction of SARS-CoV-2-specific T cells influences COVID-19 patient prognosis.

Furthermore, TCR repertoire diversity is a major determinant of disease prognosis after viral infection. Several studies have shown that highly diverse TCR repertoires play an important role in defense against a wide range of antigens (28, 29). In actuality, recent studies have suggested that SARS-CoV-2-specific TCRs are relatively diverse in COVID-19 mild disease patients compared with those in severe disease patients, suggesting that induction of T-cell immunity with diverse TCR repertoires may contribute to the suppression of COVID-19 severe disease (30, 31). Identification of functional TCR clones among these diverse TCR repertoires will lead to the possibility of CAR-T cell therapy in individuals at high risk for severe disease, such as the elderly and people with underlying conditions. Several groups have identified SARS-CoV-2-specific CD8^+^ T cell clones with high cytotoxic activity (32–34), and it is expected that a new method of preventing severe disease using CD8^+^ T cells will be established. In addition, mRNA vaccines expressing the SARS-CoV-2 spike protein, such as mRNA-1273 (Moderna, Cambridge, MA) and BNT162b2 (Pfizer, New York, NY), which induce antigen-specific T-cell responses (35, 36), are currently being used worldwide against COVID-19. They cause remarkable induction of CD4^+^ T-cell responses; rapid Th1 and peripheral Tfh cell induction after initial vaccination is associated with CD8^+^ T-cell responses and antibody induction after the second vaccination (37). Although the mRNA-vaccine-induced antibody population declines over time, it persists for at least 6 months (38, 39). The mRNA-vaccine-induced antigen-specific CD4^+^ T cell frequency remains stable from 3 to 6 months after vaccination, with a half-life of 187 days (40). In another study, the frequency of CD4^+^ T cells induced by low-dose mRNA-1273 was maintained even after 6 months (41). By contrast, the frequency of CD8^+^ T cells induced by mRNA vaccines decreases after 6 months (40).

Despite increasing evidence on the dynamics of mRNA vaccine-induced cellular immunity, it remains to be clarified which vaccine-induced T-cell responses better predict protection from disease after SARS-CoV-2 exposure. Furthermore, to respond appropriately to new VoCs, it is necessary to determine mRNA vaccine-induced T-cell cross-reactivity to them. T-cell immunity induced by infection or vaccination may be cross-reactive to mutant strains (42–45). T-cell responses to newly emerged Omicron strains have been actively analyzed. For instance, BNT162b2-vaccinated individuals, and those who had recovered from SARS-CoV-2 infection, had antigen-specific T cells that were cross-reactive to the Omicron variant spike protein (46–48). In vaccine- or infection-induced CD4^+^ and CD8^+^ T cells, exposure to the Omicron-derived spike-peptide pool, or wild-type spike-protein peptides, caused comparable IFN-γ, IL-2, and TNF cytokine production (49). Nonetheless, it remains unclear how the mRNA vaccine-induced cytotoxicity of cross-reactive T cells changes over time. To address this in the present study, we examined peripheral blood mononuclear cells (PBMCs) to estimate whether we could detect the spike-specific CD8^+^ T cells against not only wild-type (WT) strain but also Omicron BA1 by BNT162b2 vaccines. Moreover, we examined the cytotoxicity of SARS-CoV-2 spike-specific CD8^+^ T cells in BNT162b2 vaccinees based on the frequency of subpopulations simultaneously expressed GZMA, GZMB, and perforin since CD8^+^ T cells show antiviral effects mainly by secreting cytotoxic granules (50, 51). We found that the subpopulation frequency did not differ for the Delta and Omicron strains, which differ from the vaccine strain, indicating that the mRNA vaccine-induced antigen-specific but cross-reactive CD8^+^ T cells. Nonetheless, the frequency of this subpopulation decreased 3 months after the second vaccination, suggesting that highly functional antigen-specific CD8^+^ T cells are not maintained in the long term.

**Table:** 

REAGENT or RESOURCE	SOURCE	IDENTIFIER
Antibodies		
biotin mouse anti-human IgG (Clone G18-145)	BD Biosciences	Cat# 555785, RRID : AB_396120
Mouse anti-human CD154-FITC (Clone TRAP1)	BD Biosciences	Cat# 555699, RRID : AB_396049
Mouse anti-human CD154-PE (Clone TRAP1)	BD Biosciences	Cat# 555700, RRID : AB_396050
Mouse anti-human CD69-BB700 (Clone FN50)	BD Biosciences	Cat# 747520, RRID : AB_2744097
Mouse anti-human CD27-PE-Cy5 (Clone 1A4CD27)	Beckman Coulter	Cat# 6607107, RRID : AB_10641617
Mouse anti-human Granzyme B-PE-Cy5.5 (Clone GB11)	Thermo Fisher Scientific	Cat# GRB18, RRID : AB_2536541
Mouse anti-human CD137-PE-Cy7 (Clone 4B4-1)	BioLegend	Cat# 309818, RRID : AB_2207741
Mouse anti-human Perforin-APC (Clone B-D48)	BioLegend	Cat# 353312, RRID : AB_2571969
Mouse anti-human IFN-γ-Alexa Fluor 700 (Clone 4S.B3)	BioLegend	Cat# 502520, RRID : AB_528921
Mouse anti-human Granzyme A-Pacific Blue (Clone CB9)	BioLegend	Cat# 507207, RRID : AB_439755
Mouse anti-human CD4-BV605 (Clone L200)	BD Biosciences	Cat# 562843, RRID : AB_2737833
Mouse anti-human TNF-BV650 (Clone MAb11)	BioLegend	Cat# 502938, RRID : AB_2562741
Mouse anti-human CD8-BUV563 (Clone RPA-T8)	BD Biosciences	Cat# 612914, RRID : AB_2870199
Mouse anti-human CD3-BUV615 (Clone SP34-2)	BD Biosciences	Cat# 751249, RRID : AB_2875266
Mouse anti-human CD3-APC-Cy7 (Clone SP34-2)	BD Biosciences	Cat# 557757, RRID : AB_396863
Rat anti-human IL-2-BUV737 (Clone MQ1-17H12)	BD Biosciences	Cat# 612836, RRID : AB_2870158
Mouse anti-human CD45RO-BUV805 (Clone UCHL1)	BD Biosciences	Cat# 748367, RRID : AB_2872786
Mouse anti-human CD4-PE-Cy5.5 (Clone S3.5)	Thermo Fisher Scientific	Cat# MHCD0418, RRID : AB_10376013
Mouse anti-human CD16-Alexa Fluor 700 (Clone 3G8)	BD Biosciences	Cat# 557920, RRID : AB_396941
Mouse anti-human TCR gamma/delta-BV510 (Clone B1)	BioLegend	Cat# 331220, RRID : AB_2564275
Mouse anti-human CD56-BV605 (Clone NCAM16.2)	BD Biosciences	Cat# 562780, RRID : AB_2728700
Mouse anti-human CD14-BV570 (Clone M5E2)	BioLegend	Cat# 301832, RRID : AB_2563629
Mouse anti-human CD20-BV711 (Clone 2H7)	BioLegend	Cat# 302342, RRID : AB_2562602
Biological samples		
Peripheral blood Mononuclear cells from human	This paper	N/A
Chemicals, peptides, and recombinant proteins		
SARS-CoV-2 S protein, His Tag, Super stable trimer	ACROBiosystems	SPN-C52H9
SARS-CoV-2 Spike Trimer, His Tag (B.1.617.2/Delta)	ACROBiosystems	SPN-C52He
SARS-CoV-2 Spike Trimer, His Tag (B.1.1.529/Omicron)	ACROBiosystems	SPN-C52Hz
Benzonase Nuclease, Purity > 90%	MERCK Millipore	Cat# 70746
SARS-CoV-2 spike peptides	This study	N/A
BD GolgiPlug	BD Biosciences	Cat# 555029
BD GolgiStop	BD Biosciences	Cat# 554724
LIVE/DEAD™ Fixable Blue Dead Cell Stain Kit	Thermo Fisher Scientific	Cat# L23105
LIVE/DEAD™ Fixable Violet Dead Cell Stain Kit	Thermo Fisher Scientific	Cat# L34955
Cytofix/Cytoperm kit	BD Biosciences	Cat# 554714
SMARTer Human TCR a/b Profiling Kit	TaKaRa	Cat# 635016
MiSeq Reagent kit v3 600-cycle	Illumina	Cat# MS-102-3003
Peroxidase Substrate Solution B	KPL	Cat# 50-65-02
TMB Peroxidase Substrate	KPL	Cat# 50-76-02
HRP-conjugated streptavidin	Thermo Fisher Scientific	Cat# N100
Critical commercial assays		
One Step PrimeScript III RT-qPCR Mix	TaKaRa	Cat# RR600A
Software and algorithms		
FlowJo 10.8.1	BD Biosciences	https://www.flowjo.com/
GraphPadPrism version 9	GraphPad Software, Inc.	https://www.graphpad.com
SPICE 6.1	Roederer M et al., 2011	https://niaid.github.io/spice/
fastp version 0.12.4	https://github.com/OpenGene/fastp	N/A
MiXCR version 4.0.0	https://github.com/milaboratory/mixcr/	N/A
R version 4.1.1 and RStudio	R	N/A
VDJtools version 1.2.1	https://github.com/mikessh/vdjtools	N/A
Other		
BD Vacutainer CPT™ Tube	BD Biosciences	362761
CELLBANKER1	TaKaRa	CB011

## Materials and methods

### Key resources table Human samples

A total of 48 individuals (21 individuals recovered with SARS-CoV-2 Alpha-variant, 6 patients infected with SARS-CoV-2 Alpha-variant, and 21 BNT162b2-vaccinated healthy individuals) were enrolled in this study (**Supplemental Table 1**). Disease severity was categorized as severe (with any oxygen support, 93% ≥ SpO_2_) using a diagnostic guide from the Japanese Ministry of Health, Labour, and Welfare. PBMCs were isolated *via* density gradient centrifugation using BD Vacutainer CPT cell preparation tube with sodium heparin (Becton, Dickinson, and Co., Franklin Lakes, NJ), according to the manufacturer’s instructions. PBMCs were immersed in CELLBANKER cell freezing medium (TaKaRa) and stored in liquid nitrogen vapor until analysis.

### SARS-CoV-2 spike-specific antibody detection

The plasma levels of total IgG-targeting SARS-CoV-2 spike-specific antibodies were determined *via* ELISA. Recombinant spike proteins (WT: Wuhan-1; Alpha: B.1.1.7; Delta: B.1.617.2; and Omicron: B.1.1.529) were obtained from ACROBiosystems (Newark, DE). To calculate spike-specific antibody titers, 96-well plates were coated with SARS-CoV-2 spike protein and incubated overnight at 4°C. The plates were then washed and incubated for 1 h with blocking buffer, then washed again, and incubated with diluted plasma samples for 2 h at 25°C. Next, the plates were washed and incubated with biotinylated anti-human total IgG (BD Biosciences, San Jose, CA) for 1 h. The plates were then washed and incubated with HRP-conjugated streptavidin (Thermo Fisher Scientific, Waltham, MA) for 1 h at room temperature. The plates were then washed and incubated with TMB peroxidase substrate (KPL, Gaithersburg, MD) for color development. After 10 min, 2 mol/l H_2_SO_4_ was added to each well to stop the reaction. Antibody expression was measured by determining optical density at 450 nm using an Epoch 2 Microplate Spectrophotometer (Agilent, Santa Clara, CA). The antibody endpoint titer was determined using a cutoff value of 0.3.

### Flow cytometry analysis

For analyzing SARS-CoV-2 spike-specific T cells, we performed surface and intracellular cytokine staining of CD4^+^ and CD8^+^ T cells. Briefly, PBMCs were incubated in 1 ml RPMI 1640 medium containing 50 U/ml benzonase nuclease (Millipore, Darmstadt, Germany), 10% fetal bovine serum, and penicillin–streptomycin for 2 h. Next, cells were incubated in 200 µl medium with or without peptides (17-mers overlapping by 11 residues) corresponding to the full-length SARS-CoV-2 spike (**Supplemental Table 2**), at a final concentration of 2 µg/ml of each peptide, for 30 min. Thereafter, 0.2 µl BD GolgiPlug and 0.14 µl BD GolgiStop (both from BD Biosciences) were added and incubated for 5.5 h. The cells were then stained using the LIVE/DEAD Fixable Blue Dead Cell Stain Kit (Thermo Fisher Scientific), and stained with anti-CD3 (SP34-2), anti-CD8 (RPA-T8), anti-CD4 (L200), anti-CD45RO (UCHL1), anti-CD27 (1A4CD27), and anti-PD-1 (EH12-2H7) antibodies. After fixation and permeabilization using the Cytofix/Cytoperm kit (BD Biosciences), the cells were stained with anti-4-1BB (4B4-1), anti-CD69 (FN50), and anti-IFN-γ (4S.B3), anti-TNF (MAb11), anti-IL-2 (MQ-17H12), anti-granzyme A (CB9), anti-granzyme B (GB11), and anti-perforin (B-D45) antibodies. Cells were analyzed using a BD FACSymphony A5 flow cytometer (BD Biosciences). The data were analyzed using FlowJo v. 10.8.1. After gating live single T cells, based on forward scatter area and height (FSC-A and -H), side scatter area (SSC-A), live/dead cell exclusion, and CD3 staining, we separated the peripheral blood mononuclear cells (PBMCs) into CD4^+^ and CD8^+^ T cells. Subsequently, CD4^+^ and CD8^+^ T cells were further divided into memory phenotypes based on the expression of CD27 and CD45RO. For spike-specific CD4 T cells, memory cells were gated based on expression of CD154 and IFN-γ. For spike-specific CD8 T cells, memory cells were gated based on expression of 4-1BB and CD69. Expression of cytotoxic molecules and cytokines were determined by their mean fluorescence intensity.

### Flow cytometry cell sorting

Cryopreserved PBMVs from 6 BNT162b2 vaccinated donors were incubated in 1 ml RPMI 1640 medium containing 50 U/ml benzonase nuclease (Millipore, Darmstadt, Germany), 10% fetal bovine serum, and penicillin–streptomycin for 2 h. Next, cells were incubated in 200 µl medium with or without peptides (17-mers overlapping by 11 residues) corresponding to the full-length SARS-CoV-2 spike WT or Omicron strain, at a final concentration of 2 µg/ml of each peptide, for 4 h in presence of anti-4-1BB (4B4-1). The cells were then stained using the LIVE/DEAD Fixable Violet Dead Cell Stain Kit (Thermo Fisher Scientific), and stained with anti-CD3 (SP34-2), anti-CD8 (RPA-T8), anti-CD4 (L200), anti-CD45RO (UCHL1), anti-CD27 (1A4CD27), and anti-CD69 (FN50) antibodies. Antibodies of anti-CD16 (3G8), anti-TCRγ/δ (B1), anti-CD14 (M5E2), anti-CD56 (NCAM16.2), and anti-CD20 (2H7) were additional stained in the dump channels and excluded from analysis. The 200 cells gated to dump^-^CD3^+^CD8^+^CD69^+^4-1BB^+^ memory T cells based on CD27 and CD45RO expression were sorted using BD FACSymphony S6 cell sorter (BD Biosciences).

### TCR sequence

After sorting, cells were centrifuged and the supernatant was removed. RNA was purified using NucleoSpin RNA Plus XS kit (Macherey-Nagel) according to manufacturer’s protocol. TCR a/b libraries were prepared from each sample using the SMARTer Human TCR a/b Profiling Kit (TaKaRa) according to manufacturer’s protocol. The sequencing was performed on a MiSeq (Illumina) using the MiSeq Reagent kit v3 600-cycle. The sequences were trimmed and filtered with fastp version 0.12.4 (52). Filtered pair-end sequences were aligned and annotated using MiXCR version 4.0.0 (53). The cutoff for the frequency of TCR clones obtained from 200 cells was set at 0.5%. V-J junction Circos plots were generated using VDJtools (54) version 1.2.1 and R version 4.1.1.

### Statistics

Data were analyzed using GraphPad Prism 9. *P*-values were determined using the nonparametric Mann–Whitney *U* test and Wilcoxon matched-pairs signed-rank test. Correlations were calculated using a nonparametric Spearman’s rank test. Analysis and representation of T-cell function was performed using Simplified Presentation of Incredibly Complex Evaluations (SPICE) v. 6.1, provided by Dr. Mario Roederer (National Institutes of Health, Bethesda, MD).

## Results

### Characterization of antigen-specific antibodies, CD4^+^ T cells, and CD8^+^ T cells against SARS-CoV-2 VoCs induced by mRNA vaccination

**Figure 1:**
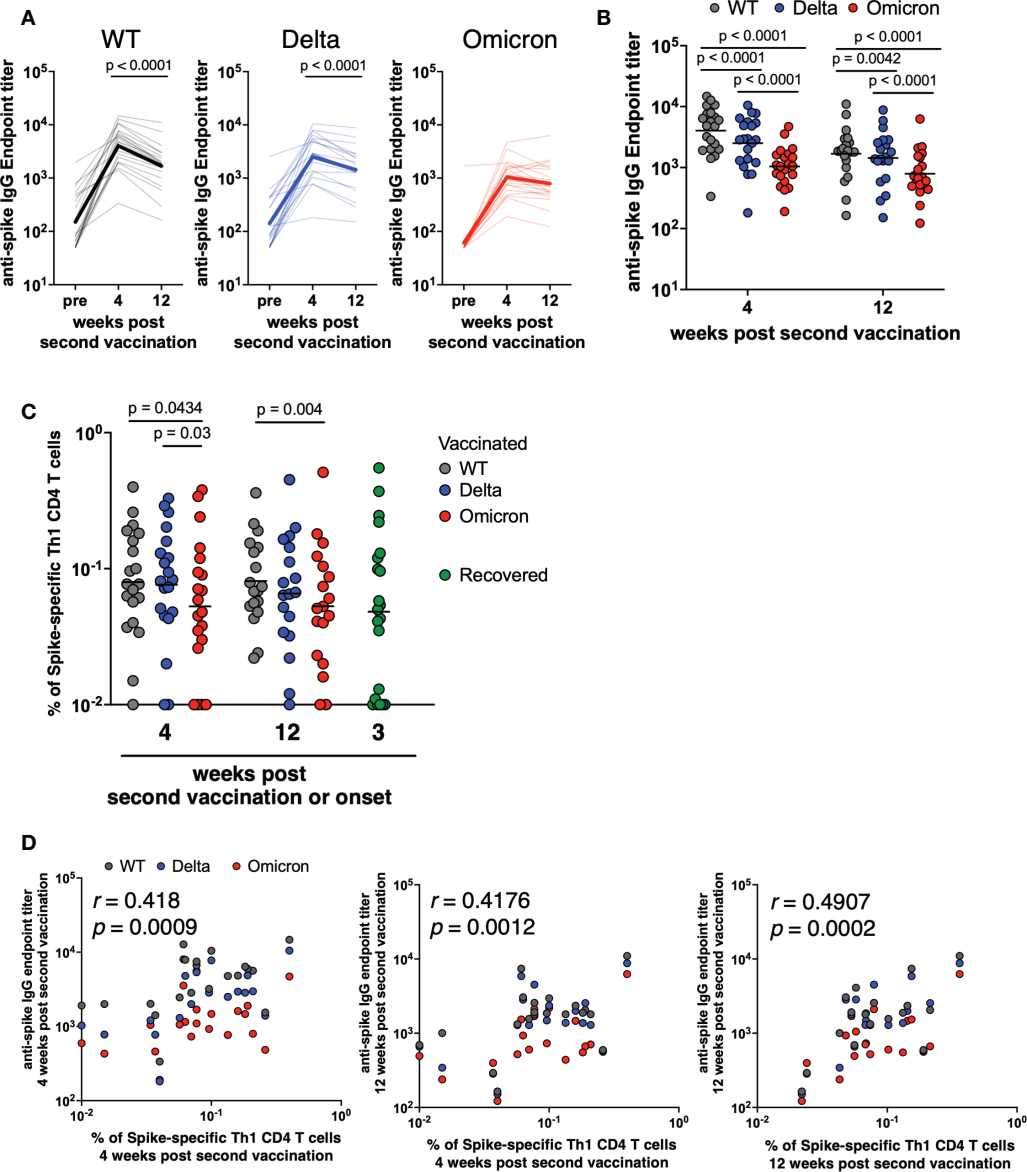
Antibody and CD4^+^ T cells induced by mRNA vaccine against SARS-CoV-2 variants of concern. **(A)** Anti-spike IgG against WT (pre; 50–1968.3, GMT = 150.2, 4 weeks; 335.7–14742.2, GMT = 4031.9, 12 weeks; 164.3–11003, GMT = 1682.7; left panel), Delta (pre; 50–2560.4, GMT = 141.9, 4 weeks; 181.6–10495.7, GMT = 2514.2, 12 weeks; 151.2–8781.6, GMT = 1435.2; center panel), and Omicron (pre; 50–1693.8, GMT = 61.1, 4 weeks; 190.5–4704.7, GMT = 1041.6, 12 weeks; 122–6260.4, GMT = 790.4; right panel) endpoint titers over time in plasma samples obtained from BNT162b2-vaccinated individuals (*n* = 21). *P*-values were calculated using the Wilcoxon matched-pairs signed rank test. **(B)** Comparison of anti-spike IgG endpoint titer against WT, Delta, and Omicron spike proteins at 4 and 12 weeks post second vaccination (*n* = 21). *P*-values were calculated using the Wilcoxon matched-pairs signed rank test. **(C)** Comparison of spike-specific Th1 cell frequency against WT (4 weeks; 0.01%–0.399%, geometric mean = 0.080%, 12 weeks; 0.022%–0.361%, geometric mean = 0.081%), Delta (4 weeks; 0.01%–0.329%, geometric mean = 0.076%, 12 weeks; 0.01%–0.451%, geometric mean = 0.066%), and Omicron (4 weeks; 0.01%–0.379%, geometric mean = 0.053%, 12 weeks; 0.01%–0.511%, geometric mean = 0.053%) spike peptides in CD4^+^ total memory cells at 4 and 12 weeks post second vaccination (*n* = 21) and spike-specific Th1 cell frequency against Alpha spike peptides in CD4^+^ total memory cells from recovered donors (0.01%–0.55%, geometric mean = 0.048%; *n* = 21). *P*-values were calculated using the Wilcoxon matched-pairs signed rank test. **(D)** Correlation of anti-spike IgG endpoint titer and spike-specific Th1 cell frequency against WT (black), Delta (blue), and Omicron (red) spike peptides (*n* = 21). Correlations were calculated using the nonparametric Spearman’s rank test.

To measure mRNA vaccine-induced immune responses against SARS-CoV-2 VoCs, we enrolled 21 healthy adults vaccinated with Pfizer BNT162b2, and obtained samples at three time points: pre-vaccination, 4 weeks after the second vaccination, and 12 weeks after the second vaccination. First, we measured the anti-spike IgG endpoint titer in response to WT, Delta, and Omicron spikes in plasma samples by performing enzyme-linked immunosorbent assay (ELISA) (*p*<0.0001 among 4 weeks vs. 12 weeks against WT and Delta; **Figure 1A**). At 4 weeks post second vaccination, antibody titers were high for all spike types (WT, Delta, and Omicron; **Figure 1A**), but lowered 12 weeks post booster vaccination (**Figure 1A**), consistent with previous reports (40, 55). At each time point, antibody titers were significantly lower in response to Delta and Omicron spikes than to the WT (*p*<0.0001 among WT vs. Delta, WT vs. Omicron and Delta vs. Omicron at 4 weeks, *p*=0.0042 among WT vs. Delta at 12 weeks, *p*<0.0001 among WT vs. Omicron and Delta vs. Omicron at 12 weeks; **Figure 1B**). In particular, 4 weeks post booster vaccination, the antibody endpoint titer was 84% lower against the Omicron spike than that against the WT.

To evaluate the role of T cells in protecting against SARS-CoV-2 VoCs exposure, we used flow cytometric analysis to examine CD4^+^ T cell responses to Delta and Omicron. PBMCs from 21 BNT162b2-vaccinated healthy donors were stimulated with WT, Delta, or Omicron strain-derived spike peptides. Spike-specific Th1 cells were defined as CD4^+^ total memory T cells expressing CD154 and IFN-γ (gating scheme shown in **Supplemental Figures 1A, B**). Spike-specific Th1 cell frequencies were calculated in stimulated samples by subtracting background of unstimulated samples: relative to the response to the WT, spike-specific Th1 cell frequency was slightly lower in response to the Omicron variant, but not to the Delta variant (*p* = 0.0434 among WT vs. Omicron at 4 weeks, *p* = 0.03 among Delta vs. Omicron at 4 weeks, *p* = 0.004 among WT vs. Omicron at 12 weeks; **Figure 1C**). Moreover, the frequency of Th1 cells induced by mRNA vaccination was comparable to that of COVID-19 recovered individuals. We evaluated the correlations between the antibody titers and Th1 cell frequencies: the Th1 cell frequency and the anti-spike endpoint titer were significantly correlated (*r*= 0.418, *p* = 0.0009 between anti-spike IgG titer and spike-specific Th1 at 4 weeks, *r* = 0.4176, *p* = 0.0012 between anti-spike IgG titer at 12 weeks and spike-specific Th1 at 4 weeks, *r* = 0.4907, *p* = 0.0002 between anti-spike IgG titer and spike-specific Th1 at 12 weeks; **Figure 1D**), suggesting that mRNA vaccine-mediated induction of Th1 cells is necessary to induce antibodies against VoCs. These results suggest that the response of antigen-specific CD4^+^ T cells is slightly lower to Omicron than to the WT; however, these cells are maintained for at least 3 months after the second vaccination.

**Figure 2:**
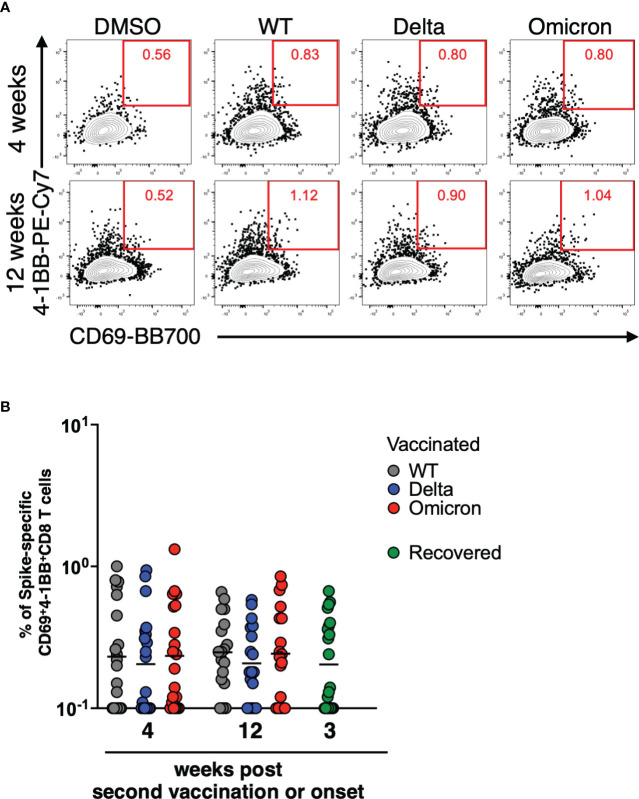
CD8^+^ T cells induced by mRNA vaccine against SARS-CoV-2 variants of concern. **(A)** After gating CD8^+^ memory T cells as in **Supplemental Figure 1A**, SARS-CoV-2 spike-specific CD8^+^ T cells were defined as the CD69^+^4-1BB^+^ population. **(B)** Comparison of spike-specific CD69^+^4-1BB^+^ CD8^+^ T cell frequency against WT (4 weeks; 0.1%–1%, geometric mean = 0.23%, 12 weeks; 0.1%–0.66%, geometric mean = 0.24%), Delta (4 weeks; 0.1%–0.94%, geometric mean = 0.20%, 12 weeks; 0.1%–0.58%, geometric mean = 0.21%), and Omicron (4 weeks; 0.1%–1.32%, geometric mean = 0.23%, 12 weeks; 0.1%–0.85%, geometric mean = 0.24%) spike peptides in CD8^+^ total memory cells at 4 and 12 weeks post second vaccination (*n* = 21) and spike-specific CD69^+^4-1BB^+^ CD8^+^ T cell frequency against Alpha spike peptides in CD8^+^ total memory cells from recovered donors (0.1–0.67%, geometric mean = 0.20%; *n* = 21) The lines show the geometric mean.

Next, we examined whether the spike-specific CD8^+^ T cells are induced by the mRNA vaccine, and maintained for a long period. The gating scheme and fluorescence-minus-one controls for CD69 and 4-1BB were shown in **Supplemental Figures 1A, C**, respectively, and the representative plots are shown in **Figure 2A**. Spike-specific CD69^+^4-1BB^+^CD8^+^ T cells were induced by the mRNA vaccine as in COVID-19 recovered individuals, and their frequencies were maintained for at least 12 weeks post second vaccination (**Figure 2B**). Furthermore, the frequency of spike-specific CD69^+^4-1BB^+^CD8^+^ T cells did not differ significantly in response to the WT, Delta, and Omicron variants, suggesting that mRNA vaccine-induced antigen-specific CD8^+^ T cells have potency of responding to SARS-CoV-2 VoCs.

### TCR repertoires of CD8^+^ T cells induced by mRNA vaccines decrease over time

**Figure 3:**
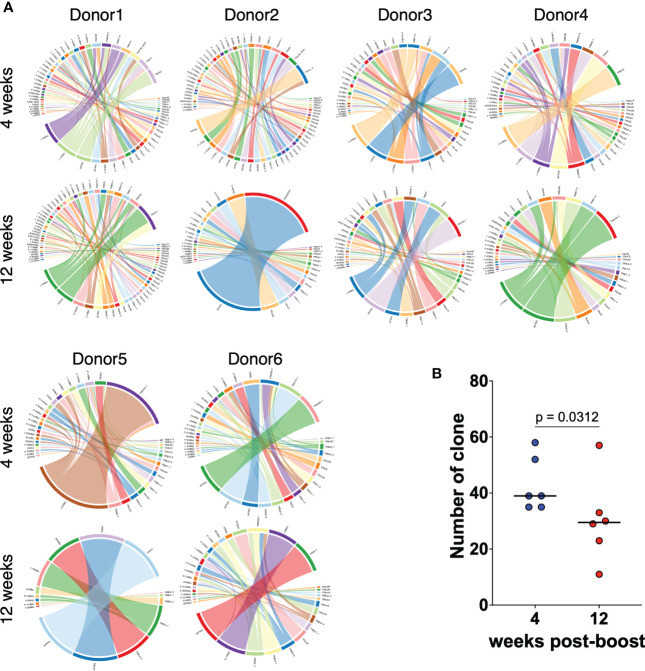
TCR repertoires of CD8^+^ T cells induced by mRNA vaccines decrease over time. **(A)** Circos plots representing the frequencies of V-J combinations in six vaccinated donors at 4 weeks and 12 weeks post second vaccination for SARS-CoV-2 WT-derived spike-specific CD8^+^ T cells. **(B)** Dot plots representing the number of TCR clones as in **(A)**. *P*-values were calculated using the Wilcoxon matched-pairs signed rank test.

We next performed TCR-seq analysis using a next-generation sequencer to determine how the TCR clonality of spike-specific CD8^+^ T cells changes over time. TCR sequences of spike-specific CD8^+^ T cells from 6 vaccinated donors were obtained by next-generation sequencing. The resulting Circos plot for each donor shows the combination of the V and J genes in the TCR at 4 and 12 weeks after the mRNA vaccine boost (**Figure 3A**). The results showed polyclonal expansion of spike-specific CD8 T cells at 4 weeks, but a decrease of TCR repertoires at 12 weeks (*p* = 0.0312 among 4 weeks vs. 12 weeks; **Figure 3B**). To compare TCR clones of spike-specific CD8^+^ T cells at 4 and 12 weeks post vaccination, amino acid sequences of CDR3 regions, V, D, and J genes and their respective frequencies were listed from the obtained sequence reads (**Supplemental Figure 2**). Although the number of TCR clones obtained was limited owing to the small number of cells analyzed, common TCR clones were detected at 4 and 12 weeks. These results suggest that the clonality of CD8^+^ T cells induced by mRNA vaccines decreases over time, while some clones are maintained over the long term.

### Spike-specific CD8^+^ T cells induced by mRNA vaccines are cross-reactive to Omicron strains

**Figure 4:**
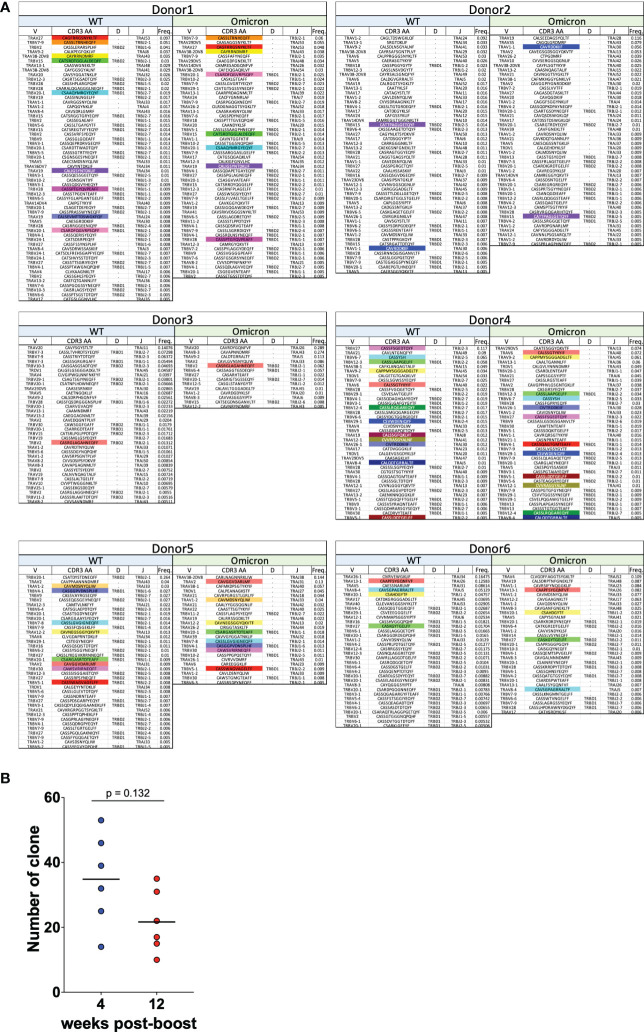
CD8^+^ T cells induced by mRNA vaccines are cross-reactive to Omicron strains. **(A)** TRAV, TRAJ, TRBV, TRBD, and TRBJ usage, CDR3 amino acid sequence, and the relative frequencies of CD8^+^ T cell clonotypes specific for the SARS-CoV-2 spike WT and Omicron are shown for six vaccinated donors at 4 weeks after the second vaccination. Colored boxes indicate CDR3 amino acid sequence reactive in common to WT and Omicron. **(B)** Dot plots representing the number of TCR clones against Omicron strain.

To verify whether spike-specific CD8^+^ T cells induced by WT-type mRNA vaccines cross-react at the TCR clone level against the Omicron strain, TCR-seq was performed on spike-specific CD8^+^ T cells responding to Omicron strain as in **Figure 3**. The amino acid sequences of the CDR3 region, V, D, and J genes, and their frequencies were listed, and the clones commonly reactive to WT and Omicron antigens in each donor were indicated by color boxes (**Figure 4A**). At least one or more TCR clones were detected in each donor in spike-specific CD8^+^ T cells against WT and Omicron strains. This suggests that mRNA vaccine-induced spike-specific CD8^+^ T cells are cross-reactive not only to WT but also to Omicron strains. Furthermore, the number of TCR clones against spike derived from Omicron strain tended to decreased over time as same as against WT (**Figure 4B**).

### mRNA vaccines induce high cytotoxic CD8^+^ T cells against SARS-CoV-2 VoCs

**Figure 5:**
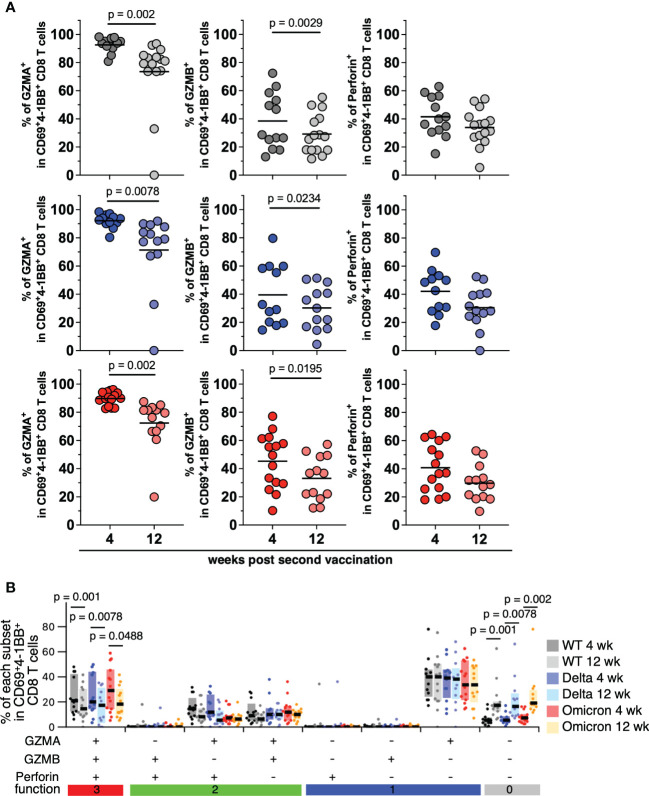
CD8^+^ T cells induced by mRNA vaccine against SARS-CoV-2 variants of concern. **(A)** Frequencies of CD69^+^4-1BB^+^CD8^+^ T cells expressing granzyme A (WT; 80.89%–98.25%, mean = 92.62% at 4 weeks, 0%–93.42%, mean = 73.56% at 12 weeks, Delta; 80.32%–98.5%, mean = 92.17% at 4 weeks, 0%–91.87%, mean = 71.4% at 12 weeks, Omicron; 82.54%–96.1%, mean = 89.77% at 4 weeks, 19.91%–87.48%, mean = 72.34% at 12 weeks; left panels), granzyme B (WT; 13.04%–72.4%, mean = 38.44% at 4 weeks, 11.63%–55.25%, mean = 29.19% at 12 weeks, Delta; 14.57%–79.65%, mean = 39.55% at 4 weeks, 4.44%–51.41%, mean = 30.21% at 12 weeks, Omicron; 10.2%–77.16%, mean = 45.3% at 4 weeks, 12.11%–57.22%, mean = 33.09% at 12 weeks; center panels), or perforin (WT; 15.22%–63%, mean = 41.51% at 4 weeks, 5.41%–54.2%, mean = 33.97% at 12 weeks, Delta; 17.84%–69.76%, mean = 42.08% at 4 weeks, 0%–52.5%, mean = 30.50% at 12 weeks, Omicron; 18%–64.4%, mean = 40.77% at 4 weeks, 9.73%–52.72%, mean = 29.77% at 12 weeks; right panels) responding to SARS-CoV-2 WT, Delta, or Omicron spike peptides (upper, middle, and lower panels, respectively; WT, 4 weeks, *n* = 13; WT, 12 weeks, *n* = 15; Delta, 4 weeks, *n* = 12; Delta, 12 weeks, *n* = 13; Omicron, 4 weeks, *n* = 15; Omicron, 12 weeks, *n* = 13). Lines show the mean. *P*-values were calculated using the Wilcoxon matched-pairs signed rank test. **(B)** Frequencies of spike-specific CD69^+^4-1BB^+^CD8^+^ T cell subpopulations expressing different combination of granzyme A, granzyme B, and perforin (WT; 6.52%–48.9%, mean = 23.43% at 4 weeks, 0%–42.1%, mean = 17.52% at 12 weeks, Delta; 4.17%–48.8%, mean = 23.5% at 4 weeks, 0%–35.5%, mean = 16.15% at 12 weeks, Omicron; 6.12%–59.6%, mean = 30.45% at 4 weeks, 6.32%–42%, mean = 19.28% at 12 weeks in all positive population). *P*-values were calculated using the Wilcoxon matched-pairs signed rank test. *P*-values were calculated using the nonparametric Mann–Whitney *U* test.

To examine whether mRNA vaccination induces antigen-specific CD8^+^ T cells with high cytotoxic activity, which can contribute to the severity of COVID-19, we analyzed PBMCs derived from vaccinees by FACS. Cytokine production by CD8^+^ T cells responding to SARS-CoV-2 spike peptides was low and difficult to quantify (**Supplemental Figures 3A, B**). To analyze antigen-specific CD8^+^ T cell cytotoxicity, we evaluated the expression of cytotoxicity-related molecules in CD69^+^4-1BB^+^CD8^+^ T cells responding to VoCs at 4 and 12 weeks after the second vaccination. The expression patterns of GZMA, GZMB and perforin were shown in **Supplemental Figure 3C**. GZMA was overexpressed in the CD69^+^4-1BB^+^CD8^+^ T cells of the vaccinated individuals (**Figure 5A**, left panels). However, relative to their expression at 4 weeks, expression at 12 weeks post second vaccination was significantly lower for GZMA (*p* = 0.002 among 4 weeks vs. 12 weeks against WT, *p* = 0.0078 among 4 weeks vs. 12 weeks against Delta, *p* = 0.002 among 4 weeks vs. 12 weeks against Omicron; **Figure 5A**, left panels) and GZMB (*p* = 0.0029 among 4 weeks vs. 12 weeks against WT, *p* = 0.0234 among 4 weeks vs. 12 weeks against Delta, *p* = 0.0195 among 4 weeks vs. 12 weeks against Omicron; **Figure 5A**, center panels). Meanwhile, there was no significant difference in perforin expression of antigen-specific CD8 T cells between 4 and 12 weeks post vaccination (**Figure 5A**, right panels). Next, we evaluated the cytotoxicity spectrum of the CD69^+^4-1BB^+^CD8^+^ T cells from vaccinated individuals. The frequency of the T-cell subpopulation simultaneously expressing GZMA, GZMB, and perforin was moderate in vaccinated individuals at 4 weeks post second vaccination (**Figure 5B**). Furthermore, at 12 weeks post second vaccination, the frequency of this subpopulation was significantly lower than that at 4 weeks (*p* = 0.001 among 4 weeks vs. 12 weeks against WT, *p* = 0.0078 among 4 weeks vs. 12 weeks against Delta, *p* = 0.0488 among 4 weeks vs. 12 weeks against Omicron in all positive population; *p* = 0.001 among 4 weeks vs. 12 weeks against WT, *p* = 0.0078 among 4 weeks vs. 12 weeks against Delta, *p* = 0.002 among 4 weeks vs. 12 weeks against Omicron in all negative population; **Figures 5B** and **Supplemental Figure 3D**), suggesting that mRNA-induced antigen-specific CD8^+^ T cell cytotoxicity is not long-lasting. By contrast, antigen-specific CD8^+^ T-cell cytotoxicity did not differ significantly among the VoCs (Delta and Omicron) and the WT strain, indicating that mRNA vaccine-induced CD8^+^ T cells cross-react with various VoCs.

## Discussion

SARS-CoV-2, which first emerged in 2019 in Wuhan, China, is still raging globally. The mRNA vaccines developed to fight this pandemic, now used worldwide, were approved in a shorter-than-usual period owing to the urgency of the situation. Although many studies on longitudinal post-vaccination have focused on neutralizing antibodies and suggested immunological correlates of protection from infection (56), phenotypic analysis of CD8^+^ T cells is essential, especially for the prevention of severe disease. We, therefore, examined the phenotype of SARS-CoV-2 spike-specific CD8^+^ T cells using TCR stimulation-dependent CD69 and 4-1BB upregulation as indicators, and found that the spike-specific CD8^+^ T cells expressing GZMA, GZMB and Perforin was decreased with overtime but cross-reacted to Omicron strain.

A recent study using PBMCs derived from vaccinees and recovered patients showed that T-cell reactivity to Omicron strain-derived spike antigen was reduced by more than 50% in 20% of the patients (57). By contrast, other studies have demonstrated that CD8^+^ T-cell responses to Omicron strain-derived spike antigens are comparable to those of WT in analyses of various types of vaccination-derived samples (46, 47, 49). In this study, we also found that two doses of mRNA vaccine induced spike-specific CD8^+^ T cells, which were also reactive to the Delta and Omicron strains. Besides, it was recently reported that some T cells induced by the vaccine suppressed viral replication of the Omicron BA.1 strain more efficiently than the Wuhan strain, the vaccine antigen strain (58). This is because the G446S mutation in the spike protein of the Omicron BA.1 strain enhances the antigen-presenting capacity of target cells, which in turn enhances the antiviral capacity of T cells. In any case, with regard to the SARS-CoV-2 spike-specific CD8^+^ T cell response, it is suggested that the virus may be able to respond to neutralizing antibody-evading viruses.

Nonetheless, it remains unclear whether CD8^+^ T cells induced by current mRNA vaccines are cross-reactive to VoC at the level of individual TCR clones. Therefore, we performed TCR sequence analysis of SARS-CoV-2 spike-specific CD8^+^ T cells to verify cross-reactivity to VoC at the single clone level in this study. Our results show that CD8^+^ T cells with the same TCR and cross-reactivity to both WT and Omicron strains were induced in the vaccinees.

Moreover, the TCR clonality of CD8^+^ T cells reacting to the vaccine antigen decreased over time; however, at least 12 weeks after the second dose of vaccine, the TCR clones detected at 4 weeks after the vaccine remained, suggesting the formation of memory immunity. In a report comparing the acute phase of infection and 6 months post infection, the number of CD8^+^ T-cell clones detected in the recovery phase was significantly lower than the number of clones detected in the acute phase of infection, with TCR clone analysis for characteristic epitopes using MHC multimers (59). In this study, we followed changes in the TCRs of spike-specific CD8^+^ T cells in vaccinated individuals and found that their clonality decreased over time, similar to natural infection, although there were still the same TCRs at 12 weeks post boost. Although the TCR clone analysis performed in this study was in a limited number of cells in a limited number of donors, three typical TCR clones (CAVMDSNYQLIW, CAVSDSNYQLIW and CASSLASTDTQYF) specific for epitope S_269-277_ (25) restricted by the HLA A*0201 were also found in HLA-matched vaccines in this study (Donor#1, against WT antigen at wk 12; Donor#2, against WT antigen at wk 4; Donor#3, against WT antigen at wk 4; Donor#5, against WT and BA.1 antigens at wk 4; Donor#6, against BA.1 antigen in wk 4). The S_269-277_ epitope is one of the major epitopes against spike antigens, and no mutations in S_269-277_ have been found in currently emerging VOCs (24, 60). Therefore, these TCR clones induced by the mRNA vaccine may contribute to the suppression of COVID-19 severity even in the case of VOCs infection. However further investigation will be needed to address this point.

Furthermore, we found that the frequency of the spike-specific CD8^+^ T-cell subpopulation, which expresses simultaneously GZMA, GZMB and perforin, was lower 3 months after the second vaccination than at 1 month, suggesting that although the mRNA vaccine induces CD8^+^ T cells that reduce COVID-19 severity, the effect may not be long-lasting. Although we could not perform polyfunctional cytotoxic analysis of spike-specific CD8^+^ T cells from COVID-19 patients with severe disease at three weeks post natural infection because of the low frequency of spike-specific CD8^+^ T cells (**Supplemental Figure 4A**), we detected polyfunctional cytotoxic CD8 T cells in the recovered donors at three weeks post natural infection (**Supplemental Figure 4B**). Expression of cytotoxic molecules in CD8^+^ T cells changes during the course of SARS-CoV-2 infection and varies between studies, as determined based on bulk analysis of T-cell responses. For instance, GZMB and perforin expression is higher in SARS-CoV-2-infected patients than in healthy donors (61), whereas another study reported that CD8^+^ T cell GZMA and perforin expression was comparable between healthy individuals and SARS-CoV-2-infected patients (62). In addition, analysis of antigen-specific CD8^+^ T cells using HLA tetramers reveals that GZMB and perforin expression levels decrease with time after COVID-19 recovery (63), and a study based on serum levels rather than intracellular expression analysis revealed that GZMA and perforin serum levels are higher in SARS-CoV-2-infected patients with mild symptoms than in healthy individuals and critically-ill patients (64). Furthermore, it has been reported that Perforin, GZMB and Granulysin expression levels were upregulated in the acute phase of mild COVID-19 patients compared to moderate and severe patients (65). More recently, analyses using MHC multimers suggest that CD8 T cells induced by the third mRNA vaccine return to pre-booster vaccination levels in ~2 months (66). Notably, the expression levels of GZMB and perforin in this epitope-specific CD8 T cells diminished over time after two doses of the vaccine and did not increase after the third dose of the vaccine. Although this report is an analysis using a typical spike epitope, it will be important to identify spike epitopes that may increase cytotoxic activity upon booster vaccination with the vaccine in the future. Besides, involvement of CD8^+^ T cells in COVID-19 pathogenesis can be elucidated by analyzing SARS-CoV-2-specific CD8^+^ T cell characteristics. In a study using HLA-A02 tetramer (60), antigen-specific CD8^+^ T cells (obtained from COVID-19 acute-phase patients) responding to the S_269–277_ epitope of the spike protein predominantly belonged to the subpopulation simultaneously expressing GZMA, GZMB, GZMK, and perforin. These studies indicate that induction of highly cytotoxic CD8 T cells may regulate COVID-19 disease progression. However, further studies are needed because of lacking direct evidence of these cytotoxic CD8T cells are associated with good prognosis.

Based on the results of multicolor flow cytometry of SARS-CoV-2 spike-specific CD8^+^ T cells in PBMCs obtained from healthy BNT162b2 mRNA vaccine recipients, the frequency of the subpopulation simultaneously expressing GZMA, GZMB, and perforin was 23.43% in CD69^+^4-1BB^+^CD8^+^ T cells 1 month after the second vaccination. At 3 months post second vaccination, this subpopulation significantly reduced to 15%. Although SARS-CoV-2 spike-specific CD8^+^ T cells were present 3 months after vaccination (based on the presence of CD69^+^ and 4-1BB^+^ T cells), their functionality declined gradually. Furthermore, consistent with recent reports (46, 47), subpopulation frequency did not differ significantly between cytotoxic CD8^+^ T cells reactive to the Delta and Omicron strains and those reactive to the vaccine strain and reactivity to Omicron strains at the clonal level based on TCR analysis, providing further evidence that CD8^+^ T cells induced by the vaccine are cross-reactive to VoCs although we could not evaluate the actual killing activity of these CD8^+^ T cells. Currently, mRNA vaccination against the Omicron strain is in progress owing to the spread of the Omicron strain (67). Since this study shows that there are TCR clones that cross-react with Omicron strains even with WT-type mRNA vaccines, a booster effect of Omicron-type mRNA vaccines on CD8^+^ T cells is expected.

Together, our findings suggest that mRNA vaccination induces a subpopulation of CD8^+^ T cells that may reduce COVID-19 severity. Nonetheless, inducing as well as maintaining this subpopulation over the long-term remains challenging. To increase the efficacy of future vaccines against VoCs, it is worth considering incorporating factors that more efficiently induce CD8^+^ T cells with highly cytotoxic activity that can remain in system for the long-term.

## Data availability statement

The data presented in this study are deposited in the DDBJ repository, accession number DRA015229. The data can be found online at: https://ddbj.nig.ac.jp/resource/sra-submission/DRA015229.

## Ethics statement

The studies involving human participants were reviewed and approved by the institutional ethics committees of the National Institutes of Biomedical Innovation, Health and Nutrition (approval no. 137 and 117-4), Osaka, Japan, and The Research Foundation for Microbial Diseases of Osaka University (approval no. 20-02), Osaka, Japan. The patients/participants provided their written informed consent to participate in this study.

## Author contributions

Conceptualization: TN and TY. Investigation: TN, KS, YM, MY, MI, YK, and AW. Resources: SM, TK, ST and YY. Writing - original draft: TN and TY. Writing - review & editing: All authors. Funding acquisition: TY. Supervision: TY. All authors contributed to the article and approved the submitted version.
