# Supplementary material for: Functional changes in cytotoxic CD8+ T-cell cross-reactivity against the SARS-CoV-2 Omicron variant after mRNA vaccination
Source: Front Immunol. 2023 Jan 4;13:1081047. doi: 10.3389/fimmu.2022.1081047 (PMC9845949; doi:10.3389/fimmu.2022.1081047)
Supplement: Supplementary file 1 [file DataSheet_1.docx]

**Supplemental Figure 1. Gating strategy for antigen-specific CD4 and CD8 T cells related to Figures 1 and 2.**

(**A**) After gating live single T cells, based on forward scatter area and height (FSC-A and -H), side scatter area (SSC-A), live/dead cell exclusion, and CD3 staining, we separated the peripheral blood mononuclear cells (PBMCs) into CD4^+^ and CD8^+^ T cells. Subsequently, CD4^+^ and CD8^+^ T cells were further divided into memory phenotypes based on the expression of CD27 and CD45RO. (**B**) Peripheral blood mononuclear cells (PBMCs) obtained from BNT162b2-vaccinated healthy individuals at four and twelve weeks post-second vaccination were either not stimulated (DMSO control) or stimulated with SARS-CoV-2 spike peptides (WT, Delta, or Omicron) for 6 h. Th1 cells were defined as CD154^+^IFN-g^+^CD4^+^ memory T cells. (**B**) After gating CD8^+^ memory T cells, SARS-CoV-2 spike-specific CD8^+^ T cells were defined as the CD69^+^4-1BB^+^ population. Upper panels show the fluorescence-minus-one controls for CD69-BB700, the middle panels for 4-1BB-PE-Cy7, the lower panels for full stains.

**Supplemental Figure 2. List of TCR clonotypes related to Figure 3.**

TRAV, TRAJ, TRBV, TRBD and TRBJ usage, CDR3 amino acid sequence, and the relative frequencies of CD8^+^ T cell clonotypes specific for the SARS-CoV-2 spike WT are shown for 6 vaccinated donors at 4 and 12 weeks after second vaccination. Colored boxes indicate CDR3 amino acid sequence in common to 4 and 12 weeks.

**Supplemental Figure 3. Functional characteristics of SARS-CoV-2 spike-specific CD8^+^ T cells from vaccinated donors related to Figure 5.**

(**A**) Frequencies of IFN-g-, TNF-, or IL-2-producing CD8^+^ T cells (upper, middle, and lower panels, respectively) responding to SARS-CoV-2 spike peptides in CD8^+^ total memory cells from BNT162b2-vaccinated healthy individuals. The lines show the geometric means. (**B**) Frequencies of subpopulations of spike-specific CD8^+^ T cells producing IFN-g, TNF, and IL-2 in cytokine-secreting CD8^+^ total memory cells. (**C**) Representative plots of granzyme A (GZMA), granzyme B (GZMB) and perforin expression in CD69^+^4-1BB^+^CD8^+^ memory T cells from patients. The gray contour plots represent the expression of each cytotoxic molecule in CD8^+^ naive T cells. (**D**) Frequency of highly cytotoxic spike-specific CD69^+^4-1BB^+^CD8^+^ T cells from vaccinated donors against WT, Delta and Omicron-derived spike peptides expressing granzyme A, granzyme B, and perforin. P-values were calculated using permutation tests.


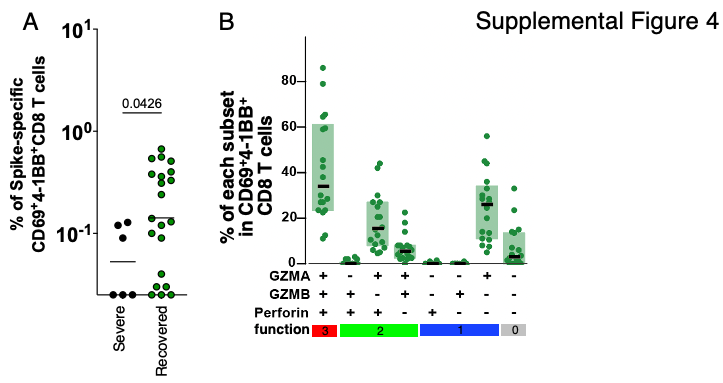


**Supplemental Figure 4. Functional characteristics of SARS-CoV-2 spike-specific CD8^+^ T cells from recovered donors.**

(**A**) Comparison of spike-specific CD69^+^4-1BB^+^ CD8^+^ T cell frequency against Alpha spike peptides in CD8^+^ total memory cells from recovered donors (*n* = 21) and COVID-19 severe diseased and unvaccinated patients (*n* = 6). The lines show the geometric mean. (**B**) Frequencies of spike-specific CD69^+^4-1BB^+^CD8^+^ T cell subpopulations expressing different combination of granzyme A, granzyme B, and perforin in recovered donors (*n* = 18).
